# How will better data (and better use of data) enable us to save the planet?

**DOI:** 10.1371/journal.pbio.3002689

**Published:** 2024-06-04

**Authors:** Frank Hawkins

**Affiliations:** International Union for Conservation of Nature, Gland, Switzerland; Arizona State University, UNITED STATES

## Abstract

The potentially catastrophic loss of biodiversity happening around the globe is largely caused by economic activity that is not connected to its environmental impacts. To improve positive and reduce negative outcomes for nature, investment decision-makers in companies, governments, and the finance sector need data on the impacts of economic activity, especially production of food and other commodities, on biodiversity, at fine geographical scales. This Essay argues that the data allowing us to identify the most important factors causing biodiversity loss are already available. However, we need more data to track impacts on biodiversity up value chains into pathways, toolkits, and approaches that will facilitate verified, concrete actions by companies and consumers to reduce threats to biodiversity in particular places. Our current knowledge is insufficient to deliver complete responses to the biodiversity crisis, but this is no excuse for delaying action.

## Abstract 2

Biodiversity is being lost because of economic activity that has no connection its impacts on biodiversity. This Essay argues that we need better spatially explicit and actionable data on the causes of biodiversity loss that can be conveyed to company decision-makers in a format that they can use easily and efficiently.

## Introduction

As a biologist working in conservation, I inhabit a community that is torn between 2 perspectives. One is that we are hurtling helplessly down a well-polished slide to biodiversity Armageddon. The other is that we are finding pathways off to the side of the slide that prevent the worst of the losses and enable humanity to persist as chastened, humble partners in a much-modified, but still viable, ecosphere.

**Fig 1:**
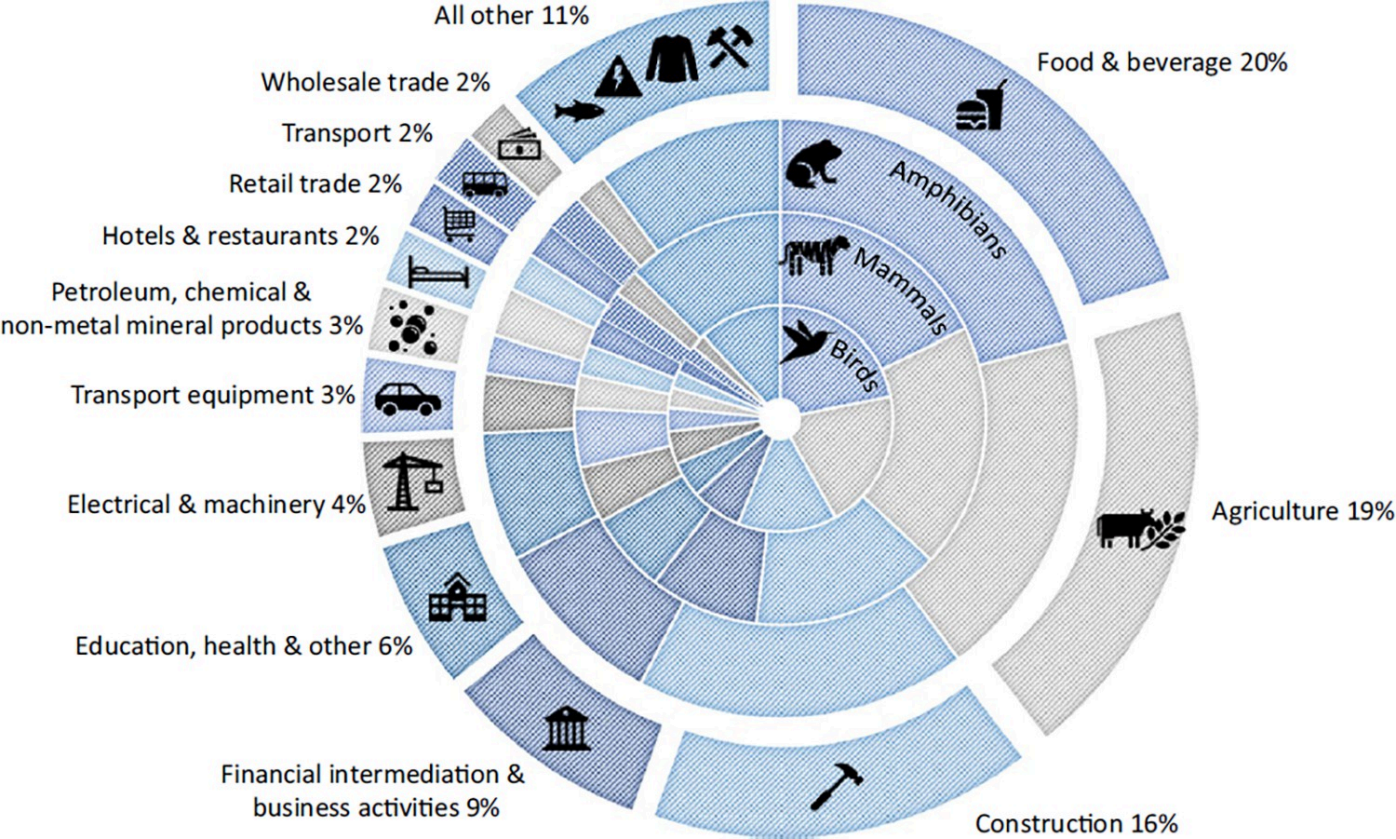
Global extinction-risk footprint by consumption sector. Source: Irwin and colleagues [8]. Licensed under Creative Commons Attribution 4.0 International License.

I oscillate between these perspectives, as do many of my colleagues. There are many very inspiring stories of individual conservation successes, often delivered in difficult and dangerous circumstances. But however positive I am, and whatever solace is derived from knowledge of these successes, one particular thing bothers me endlessly. That is the behavior of companies, and in particular the relentless and often unconscious asset-stripping and despoilation of nature in which we all participate through our use of these companies’ products. Of the 5 major drivers of biodiversity loss [1], the majority of the impacts of land-use change, climate change, pollution, and natural resource use on biodiversity are driven by consumption of food mediated through companies (**Fig 1**).

Decisions about how and in what to invest, taken under cover of profit-making mandates to shareholders, lead to often inadvertent but appalling destruction and loss of nature, the impacts of which are comfortably insulated (at least in the short term) from those making the decisions. If we are to deliver the biodiversity components of the Kunming-Montreal Global Biodiversity Framework (KMGBF [2]; **Box 1**)—notably those relating to species extinction risk reduction and ecosystem conservation (Goal A)—massive realignment of financial flows is unavoidable.

**Box 1. Relevant goals and targets from the Kunming-Montreal Global Biodiversity Framework**

**2050 Goals**

Goal A: The integrity, connectivity, and resilience of all ecosystems are maintained, enhanced, or restored, substantially increasing the area of natural ecosystems by 2050. Human-induced extinction of known threatened species is halted, and, by 2050, the extinction rate and risk of all species are reduced 10-fold and the abundance of native wild species is increased to healthy and resilient levels. The genetic diversity within populations of wild and domesticated species is maintained, safeguarding their adaptive potential.

2030 Targets

**TARGET 1: Plan and manage all areas to reduce biodiversity loss**

**TARGET 2: Restore 30% of all degraded ecosystems**

**TARGET 3: Conserve 30% of land, waters, and seas**

**TARGET 4: Halt species extinction, protect genetic diversity, and manage human–wildlife conflicts**

**TARGET 15: Businesses assess, disclose, and reduce biodiversity-related risks and negative impacts**

**TARGET 19: Mobilize $200 billion per year for biodiversity from all sources, including $30 billion through international finance**

This paper brings a personal opinion about how to acquire and deploy data that stand the best chance of influencing how decisions around financial flows can be made that minimize the worst impacts on biodiversity, and conversely maximize the positive impacts. Such is the recent flowering of metrics and indicators relating to biodiversity that it would take several papers of this length to provide a comprehensive analysis of the status quo—such reviews already exist [3,4,5]. Instead, I have tried to focus on the general requirements that new data and metrics need to have in order to remedy this situation [6], while using some examples of my own work within International Union for Conservation of Nature (IUCN) as illustrations. These examples should not, therefore, be seen as representing the whole of a complex and rapidly moving world of research.

From the perspective of a biologist, the clear issue is that we do not have enough data that links the global economy with biodiversity. As an example, I’ve studied the local-level impacts of logging on birds in western Madagascar [7] and my colleagues and I can link the details of how these forests are managed to the likelihood of species extinctions. However, tracking these impacts through the full range of activities required to take a wood product from harvesting and processing to consumption and disposal—known as the value chain—so that the people who are buying the timber can understand how their purchase affects species, and from there the outcomes of the KMGBF (**Box 1**), is very difficult. Some aspects of the impacts of forest management including clearance for agriculture (a more serious problem) on biodiversity in Madagascar have been explored, but only at a general level [8]. Multiply this issue by 10,000, as a guess at the number of ways in which value chains interact with ecosystems world-wide, and the scale of the problem becomes apparent. If the KMGBF is to succeed, what do we need to do, in terms of acquisition of information and use of that data, to influence investment decisions?

Given the scale of the flows of money, the number of ways in which these flows interact with biodiversity, and the number of disconnected, stressed, and financially motivated people making investment decisions, it’s hard to see how to have the necessary scale of change. Advocates of systemic revolution in the finance system I feel are deluding themselves [9], although the more protest and agitation pointing out the problem the better [10]. But short of revolution, what else is there?

### Increase investment in biodiversity

Our collective efforts up to now have focused on 2 approaches to alter how companies interact with nature. Firstly, opening the door to profit-making through investment in biodiversity [11]. This seems like an ideal solution, on the face of it. Not only can it deliver positive biodiversity outcomes, if projects involve people with limited economic opportunities, then building new development options for them helps alleviate poverty too. Constructing investment opportunities to deliver positive biodiversity outcomes has precedents—through companies that produce certified commodities, ecotourism operations, and more recently biodiversity credit markets [12]. Investor motivation for participation in these markets is varied (**Box 2**) and may be more related to positioning and image than pure profit-seeking [13].

**Box 2. Biodiversity credits**

Business needs influencing demand for biodiversity credits:

- Enhanced environmental, social, and governance (ESG) credentials
- Storytelling to stakeholders
- Employee values
- Real ecological impact
- Affordability
- Forestalling regulation
- Preemptive minimization of public relations crises

However, the practical difficulties of putting together a project that genuinely delivers positive biodiversity outcomes, while resolving resource governance and equity issues that are often tangled and complex matters in countries rich in biodiversity, and then generating a risk-adjusted rate of return, are immense [12]. Bringing them to the scale required by institutional investors—which involves assembling these usually small deals into finance-sector friendly multimillion dollar vehicles—is also a huge impediment, a cost that the finance sector won’t cover. While the net amount of money going to nominally environmental projects has increased, environmental bonds are in particular seeing a tremendous amount of growth [12], few (less than 10% in 2023) are biodiversity labeled funds, and almost all of these focus on ecosystems in the global north rather than on places with globally significant biodiversity [13]. The pipeline of “real” deals is still vanishingly small compared to the amounts of money that go into even small components of environmentally destructive activities such as mining or hydrocarbon extraction. The global financing gap [14] between needed and available funds, of around 500 billion to 1 trillion USD per year, is far from being filled by private finance [15] and governmental expenditure on subsidies is currently about 2 to 4 times higher than public financing of conservation [16].

While the impediments to scaling private investment in conservation are clear—investors are always on the hunt for cheap and easy returns and most conservation projects have a poor risk return profile, small ticket sizes, and high set-up costs [16]—the solutions have not yet been deployed at scale. Why not remove some of the risk for investors by providing concessional or grant finance, first-loss funds, and credit guarantees? Persuading development finance institutions to put up the sums required in risk finance to make the deals more palatable has been very painful [16]. It seems to me that using development finance in this way to increase private investment in a global public good of such fundamental importance as biodiversity is the perfect use of public money. Local communities benefit from participation in new and potentially sustainable economic activities, money flows to hitherto disengaged or even disenfranchised members of society (often also the poorest), and the world gets more and better nature. There is certainly a chicken-and-egg issue here because, where there is little project pipeline, there is little demand for the risk finance, and thus little risk finance available. While the steps to delivering the solutions to this problem are evident [16], we’re not seeing much in terms of delivery. When we do, of course, it will be essential to ensure that the massive investments required actually deliver their promised positive impacts for biodiversity, an outcome that is currently hard to demonstrate, even for conservation biologists. Investors recognize that the lack of an appropriate measurement framework that guides action is a barrier to investment [12]. However, there is little motivation, and hitherto no practical mechanism, to measure the loss of biodiversity that relates to specific impacts in specific places; provides companies with a pathway to address these impacts; and delivers quantified contributions to the KMGBF biodiversity targets.

### Understanding biodiversity risk

Given the challenges with scaling up biodiversity-related markets, the second option we have to change how companies interact with the environment focuses on biodiversity-related risk. These risks fall into 2 categories termed dependency risk and impact risk. Dependency risk is where the loss of nature (e.g., insect pollinators) reduces a company’s ability to produce the products it sells, while impact risk is where the company’s actions, such as cutting down rainforest to make space for agriculture, impose costs on the rest of society through the loss of biodiversity [17].

The first task is to identify the impacts the bad corporate behavior is having on biodiversity, and what risk this exposes a business to. That, of course, means knowing where and how the underlying biodiversity is being impacted. For companies that have landholding assets over which they can exercise management authority, knowing what biodiversity risk exists is relatively simple. Tools such as the Integrated Biodiversity Assessment Tool (IBAT) [18] allow companies to evaluate the scale and nature of threats to biodiversity that might be linked to their actions at a site scale. Regulatory frameworks, such as the Performance Standard 6 of the IFC [19] and other variants used by other banks, allow the finance industry to ensure that investments are oriented to have the lowest practicable impact on biodiversity. However, there is still a big gap in commitment. A key finding [20] is that while awareness of the importance of biodiversity among asset managers has improved, there are few commitments to biodiversity and biodiversity-related risk assessments are inadequate.

Probably a more significant challenge is in linking the production zones of agricultural commodities to their use in value chains. Existing risk assessment tools such as the WWF Biodiversity Risk Filter [21], Trase [22], Forest IQ [23], ENCORE [24], Exiobase [25], and the Nature Risk Profile [26] use aggregated data on sector impacts on biodiversity, generally from underlying data sources that are at best available at country level or higher. Results of analyses using these assessment tools give companies a high-level view of where they might be exposed to risk, but do not really help in identifying actions that they can take to minimize the risk, and therefore they may not help a great deal in stemming the loss of biodiversity. An obvious challenge is to refine these models by helping companies identify in more detail where production occurs and, therefore, what impacts are happening to the underlying biodiversity. So how practical is this?

**Fig 2:**
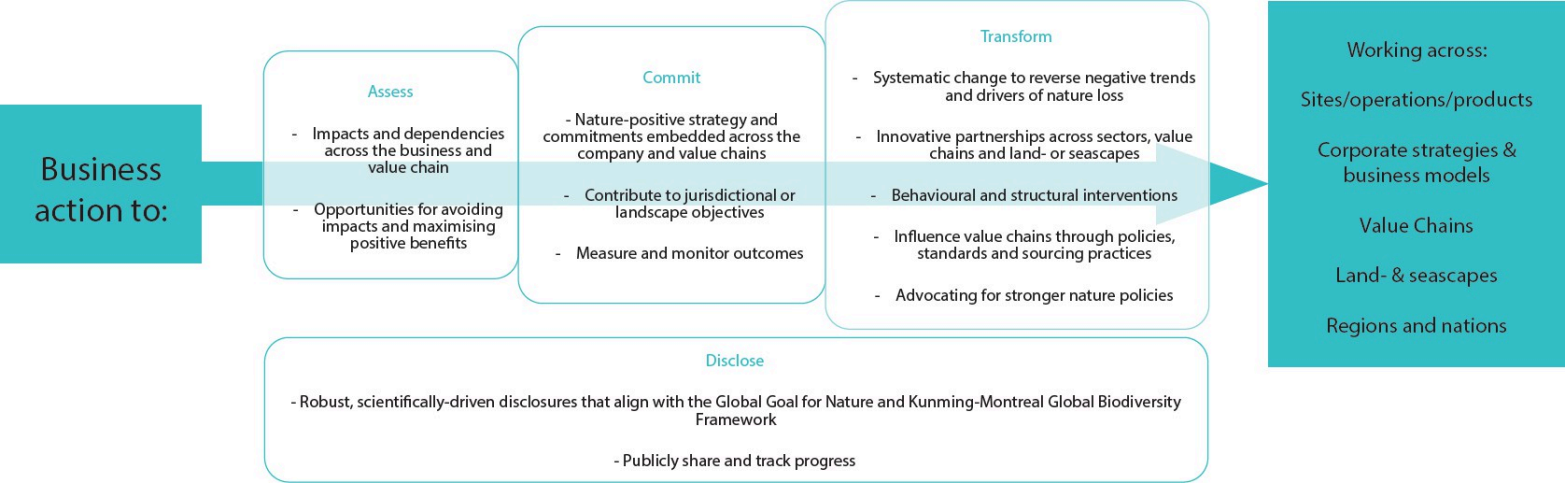
Conceptual framework for business action to contribute from the KMGBF. Adapted from Baggaley and colleagues [30].

The first step is to link the production of commodities in particular locations with impacts on biodiversity. This requires data on commodity production, for example, that available through Trase [22]. For biodiversity impacts, it is essential that biodiversity metrics are generated from “bottom-up,” spatially explicit data sets, so that the impacts of production can be tied accurately to the underlying biodiversity. One such metric is Species Threat Abatement and Restoration (STAR) [27], which can be used to quantify what the likely impact of commodity production is in certain local areas. An overall analysis of the impact of commodity production on species extinction risk globally, at a subnational and even producer level, is feasible, but it would be laborious and require in particular significant effort to link the definitions of commodities in different databases. Following this, using the information about the local manifestation of threats to species contained within STAR can allow companies to verify the presence of threatened species and ecosystems, confirm the presence and severity of threats, identify actions, set targets to reduce threat intensity and deliver validated, quantified contributions to the KMGBF (**Fig 2**). This process is described in more detail through the IUCN Measuring Nature Positive initiative [28], and this is currently being improved to enable companies to use it efficiently. The Measuring Nature Positive initiative builds on and complements the Science-based Targets Network (SBTN) [29] approach, which is currently focused, as far as target-setting is concerned, on corporate zero land-use change in the terrestrial realm and on nonliving aspects of nature in freshwater.

However, understanding where company actions have impacts on biodiversity on the ground and planning actions to mitigate threats caused by these actions is one thing. Tracking it along notoriously obscure value chains is another. There is a genuine issue for end-product companies that moving through the various tiers of suppliers for many commodities is time-consuming and expensive. Many companies say they can’t afford the time and resources necessary to work down through these tiers to where the commodity is produced. Passing the costs of obtaining sourcing data on to price-sensitive consumers will reduce demand for certified products. Increasing the costs of commodities is strongly and reasonably resisted by governments if loss of market share is a possible outcome. Another significant impediment to detailed disclosure is that companies, particularly smaller ones, feel that their sourcing practices are confidential business information. Even if companies find that their producer sources are behaving badly, the transaction costs of switching suppliers to a more responsible one are high and often accompanied by worries about sustaining supply at an appropriate level of quality. Even in cases where a company knows in detail where a producer is working, they may only be buying a small amount of product and, thus, do not have much leverage to change the production system. This may require companies to develop partnerships with other buyers of the commodity in order to induce the required action by producers.

While these impediments are currently dampening enthusiasm for voluntary disclosure, at some point soon more stringent disclosure and reporting requirements may force companies to increase their knowledge of how the commodities they use affect biodiversity. Frameworks such as the Taskforce for Nature-related Financial Disclosure [31] (TNFD) have opened companies to the notion of disclosure, with the future adoption of TNFD-based regulatory frameworks by government a strong motivator for adoption. If, as seems possible, the TNFD becomes part of the International Sustainability Standards Board [32], then as long as TNFD has the appropriate requirement for a core metric relating to biodiversity, the impact on corporate behavior could be very significant. Currently, however, there is only a “placeholder” core metric on species extinction in TNFD. If we want to ensure that corporate disclosure really relates to impacts on biodiversity, it is essential that this placeholder is replaced with a metric that genuinely reflects risk and impact to threatened species in particular places, such as STAR. In a similar way, the KMGBF currently only includes a target to “encourage” businesses and financial institutions to disclose their impacts and dependencies on biodiversity, and the weak wording implies voluntary and inconsistent disclosure that does not link directly to underlying biodiversity [33].

Companies that work with TNFD to pilot the approach say that even the current formulation is onerous, so any additional effort required to identify and act on knowledge of biodiversity impacts needs to be as simple and automated as possible.

An additional key component of making disclosure generate significant reductions on biodiversity impacts is to ensure it is backed by regulation [33]. In some places, particularly the European Union, this regulatory framework is developing. The EU European Sustainability Reporting Standards (ESRS) E4 [34], in particular, the Digital Product Passport [35], could provide a way for biodiversity impacts embodied in a particular product to be tracked through the production and consumption process and enable consumers to take a more active role affecting production standards (**Box 3**). The EU Deforestation Law [36], passed in 2023, banning imports of commodities linked to deforestation, is also likely to reduce the impact of key commodities such as cocoa, coffee, palm oil, and soy on biodiversity. However, it does also seem to be having the unfortunate impact of encouraging buyers to switch their sourcing from smallholders [37], who are less able to guarantee being deforestation-free, to larger producers who are able to make those commitments. This means that the smaller producers, faced with a lack of market for their product, turn to other means of generating livelihoods—such as subsistence agriculture—that lead to greater loss of native forest.

**Box 3. A new European framework for corporate reporting on biodiversity impact**

The EU European Sustainability Reporting Standards (ESRS), adopted in July 2023, and in particular, E4 focuses on biodiversity and ecosystems and represents an unprecedented intensification of reporting requirements that sets a standard for other regional economic bodies to follow. Only large listed companies, banks, and insurers will need to report this year, other large companies in 2025 and small- and medium-sized companies (SMEs) only in 2026, with an opt-out possible for 2 years after that. One concern expressed by many reviewers was that reporting companies are not required to justify what they consider to be material risk, and could therefore simply exclude impacts for which they have no detailed information, for instance, those generated through complex value chains.

The fundamental issue with these approaches is that the most important immediate concern of companies, reflected in the emphasis in the various disclosure frameworks, is on their dependency risk because it imposes a cost on companies and is therefore “material” to their business. This concern is reflected in how the UN System of Environmental-Economic Accounting [38] framework focuses, for governments, on how economic performance is dependent on ecosystems, not on how economic activity impacts ecosystems and species. While evaluation for dependency risk is still incomplete and reliant on models that are variably robust, there is a great deal of existing work and toolkits that help understanding of ecosystem flows and their interaction with corporate value [39,40,41]. Similarly, TNFD, for example, is focused on KMGBF target 15, improving compliance with reporting on risks, dependencies, and impacts, and not on a mechanism that allows the impact of the actions that companies take, in particular through mitigating impacts, on KMGBF targets 1 to 4 [33]. While the EU ESRS is explicitly focused on “double materiality,” requiring companies to report on both impacts and dependencies, the regulation is currently not well developed in terms of guidance to understand impacts on underlying biodiversity. Even in the KMGBF, Target 15 only requires companies to improve reporting and disclosure actions, and has no connection to delivery of Goal A or Targets 1 to 4 that refer to the underlying biodiversity. This seems likely to encourage companies not to quantify or act on their impacts on biodiversity, as reporting and disclosure actions may be seen to be sufficient [33].

A positive impact of these reporting and disclosure initiatives is that companies are familiarizing themselves with issues relating to biodiversity and recognizing that their dependency on nature is not only material, but manageable. In contrast, impacts on biodiversity imposed by companies fall on wider society and are generally not considered material, and will be only under what many companies view an extreme regulatory framework that creates materiality through compliance risk.

The insurance industry and the pensions sector are pushing for better appreciation of impact risk [42]. Insurance companies are more exposed to impact risk compared to individual producers or consumer goods companies by virtue of thinking strategically and long-term about where, and how, risks manifest themselves across all their insured companies. They can, therefore, price insurance more highly for those companies who do not manage their impact risk. The pensions sector has a similar long-term, wide-ranging view, and wants to know that its’ investees are going to be delivering the same kind of return well into the future. If companies do not reduce their biodiversity impacts they are likely to be hit by systemic risk, and those that don’t manage this increased risk are prime targets for divestment.

In order for influential sectors such as pensions and insurance to get a proper grip on the wider, systemic impacts of biodiversity loss, they need to know which companies are likely to have significant impacts on biodiversity, and based on this knowledge, are acting to resolve them.

### Metrics of biodiversity

In order to reorient flows of finance appropriately, the sources of the flows must be held accountable for their impacts on biodiversity. This means knowledge of the spatial distribution of biodiversity at fine scales reflecting the variation in distribution of species and ecosystems must be linked to the activities funded by the finance flows. A key data requirement is, therefore, to develop metrics of biodiversity that offer clear pathways to reducing the risk to the companies as well as the impacts on biodiversity. Many have tried to develop such metrics: it’s a crowded field [3,4]. Some offer a cloak of credibility to disingenuous companies through spurious claims of a “footprint” that they have remediated or conserved. This approach has been widely adopted, especially by the finance sector. The better “top-down [6],” modeled footprinting methods used in the risk assessment tools mentioned above, can enable companies to identify likely sectors, and potentially regions, where they should start acting. But in order for the rapid loss of biodiversity to be prevented, specific changes need to happen in particular places, resulting in the improved status of identifiable components of biodiversity.

Biodiversity metrics must therefore be built from the bottom up [6] and with spatial precision appropriate to the scale in variation of the underlying biodiversity. They must also indicate how the management will affect the threats to biodiversity at the same scale. At IUCN we’ve been working, with corporate, NGO, and government partners, to develop an approach known as Measuring Nature Positive [28] that allows companies to do this, and we are piloting examples of companies with specific land-based assets where defining and implementing remedial action is relatively simple. We use the Species Threat Abatement and Restoration metric to identify opportunities to reduce species extinction risk at particular places, but the framework allows aggregation upward into sectors, corporate footprints, national commitments, and ultimately the KMGBF. At present, the Measuring Nature Positive [28] approach appears to be the only framework to allow companies to identify what fraction of KMGBF biodiversity Target 4 (on species extinction risk; **Box 1**) they could deliver, in a quantifiable and verifiable manner. But it currently relies on data from species in only 3 of the animal classes mammals, birds, and amphibians, with marine and freshwater species in the close pipeline. In order for this approach to cover a larger range of biodiversity, more data is needed to: extend the species included into plants and invertebrates, review the threat status of many species, and come up with a global threat assessment of ecosystems coupled with a detailed map of their distributions.

### Technologies for data collection and provision

The use of new technology such as AI, e-DNA, camera-trapping, and auditory sampling show significant promise in improving our ability to verify species presence and confirm levels of threat abatement [43]. However, they are currently limited by the availability of reference material (especially for e-DNA) and bottlenecks in data verification. Additionally, if AI is used to generate bad-faith data, this will be both difficult to detect and will obscure real data patterns.

One business model for the provision of data of this kind—used by IBAT and many commercial providers of technologies, such as remote sensing and e-DNA—is that users pay at the point of delivery or sign up to a subscription model. For species data, this is currently the only way to ensure that even part of the cost of curation and upgrading of the data can be covered, as the philanthropic and government finance sectors seem curiously unwilling to support such crucial global public goods. If these unwilling constituencies are persuaded to provide financial support, access should be at no cost at the point of delivery and without constraint on the use of the data for decision-making by different constituents. The latter conditions are to combat a worrying trend where large data consolidators invest in data providers with the promise of support for complex and hitherto expensive analysis but, once the analysis is complete, restrict access to the products of the analysis either behind firewalls or under use restrictions that limit application to only those that the consolidator considers viable business opportunities.

To make the KMGBF achievable, it needs to be disaggregated into the contributions necessary across sectors, governments, companies, portfolios, policy options, value chains, and ultimately consumer products. Each potential contributor will need to be able to understand the exact actions that will be needed to deliver a contribution, and be able to plan and implement those actions and report on the outcomes. This will require a very substantial and robust support and analysis structure including: underlying data sets to ensure that potential contributions are assessed everywhere across the globe; sufficient bandwidth to deal with thousands of simultaneous contributions; and all the calculations and data input that would be required for each.

The value of making these data freely available goes beyond just making sure that companies are able to uphold their reporting and disclosure obligations. If governments are motivated to manage the impacts of consumption through supporting the transition to more sustainable agriculture in producer countries—via incentives or support to companies willing to help—the possibilities for change are immense. If financial vehicles of sufficient scale and risk/return profile that generate verified, sustained positive impacts on biodiversity are developed, the market could open a huge opportunity for positive finance to flow. It’s a big “if,” however.

The burgeoning interest in biodiversity credits is a clear marker that the investor interest is there (**Box 2**). But it’s currently quite difficult to nail down how demand for biodiversity credits would work, and there are substantial issues with metrics of biodiversity impacts, which need to be applicable wherever the investment is made around the world and permit comparison between metrics in an objective and scientifically robust fashion. If those conditions can be met, one of the most likely scenarios is that biodiversity credits will allow companies to make contributions to the KMGBF that relate to historic, unquantifiable or non-remediable impacts. Examples might include historic damage done to biodiversity through the purchase of commodities that were not traceable to a particular supplier, but was suspected of having caused loss. In order for these kinds of impacts to be included in a credits market, the buyer will have to seek positive impacts, for example, reduction in species extinction risk. Metrics such as STAR can be used to make these contributions as close in character as possible to the original impact.

### Data and knowledge needed to support increased investment in conservation

Where intact biodiversity has a sufficiently stable and significant value, investment in conservation can be the basis for development pathways that generate long-term and equitable revenue to indigenous people and local communities (IPLCs). If such a transition is to be achieved, these constituencies must be able to agree with investors a common framing of what intact biodiversity means. Agreements between investors and IPLCs must be concluded from a position of equal negotiating power, so that the abuses and iniquities of the past can be prevented. Knowledge of what communities mean by biodiversity assets, as well as the specific means by which those assets are to be managed, and to whom the benefits of this management accrue, must all be freely available.

Real innovation in financial vehicles, such as targeted and verified biodiversity credits, bonds, debt swaps, and results-based sovereign debt instruments, is needed to deliver conservation outcomes, using the data and metrics developed above, but also adapted to reflect interests and capacity of IPLCs and other less-well represented constituents. These will require enhanced support to small-scale actions in capacity building for local project developers and local financial institutions, who may have difficulty conducting due diligence on conservation deals with which they are unfamiliar. Above all, building this stream of investment finance requires ready access to large amounts of grant and concessional finance that can bring project developers closer to delivering investable deals into the market. These tools and approaches should be targeted at local communities, indigenous peoples, and SMEs that stand the greatest chance of delivering significant contributions to the KMGBF biodiversity targets, assessed according to the approaches considered above.

The ideal resulting situation would be a data management system that allows these disparate constituents to be referring to the same metrics for project identification, screening, risk assessment, baseline setting, target setting, and delivery of contributions to the KMGBF. TNFD and partners have proposed a Global Nature Data Facility [44]—which, if implemented appropriately including through integration of such core biodiversity data sets (and their governance mechanisms) as the Red List of Threatened Species [45], the Key Biodiversity Areas database [46], and Protected Planet [47]—could deliver this outcome. There are considerable hurdles to overcome before such an idea becomes reality, including figuring out complex and potentially divisive governance issues, data access and ownership rights, and financing, but if these issues can be resolved, and they are combined with applications such as the IUCN Measuring Nature Positive approach potential contributors will have a pathway to plan, implement, and report on their contributions.

### Conclusion and future outlook

When trying to solve any problem, there is a danger of acting too soon with insufficient information to have the desired impact, or conversely waiting too long for more data so that any actions are too late to change the outcome. Given that we already have data sets that relate particular species to the threats caused by human activities in specific locations, we know where and how to start acting to reduce these threats and therefore the impacts of some of the most important economic drivers of biodiversity loss. More data on the impacts of many commodities at fine scales, and of individual and company participation in key value chains is required. We also urgently need clear guidance and simple, automated toolkits to help companies to identify and deliver verified contributions to the KMGBF, and more precise and explicit connections between the disclosure, reporting and changes in company behavior and the delivery of these contributions. It is my view that we stand the best chance of early success leading to wider impact if we take some urgent actions now to tackle the problem of biodiversity loss (**Box 4**), and refine and reorient these actions later once further data and analyses become available.

**Box 4. Take-home messages**

- Existing biodiversity data relates species and ecosystems distributed in specific areas, 2 components of underlying biodiversity, to the threats caused by commodity production and other human activities in the same areas.
- Companies need to be aware, via efficient, accessible and relatively low-cost mechanisms, of the impacts of their actions in different locations.
- The bottleneck is not in the availability of biodiversity data; it is how we use the data we have to follow impacts up the commodity chain, and the pathways, toolkits, and approaches we use to facilitate verified, concrete actions to reduce threats to biodiversity.
- Corporate disclosure, reporting, risk evaluation, and investment strategies need to include the impacts of actions to reduce threats to underlying biodiversity.
- Increasing investment in deals that deliver positive outcomes for biodiversity has proven hard to scale and costly to deliver, but is a vital part of building sustainable economies.
- Public finance and philanthropy should be oriented towards funding new and existing data analyses, toolkits, and platforms, and supporting biodiversity-positive investment.

## Abbreviations:

ESRS: European Sustainability Reporting Standards
IBAT: Integrated Biodiversity Assessment Tool
IPLC: indigenous people and local community
IUCN: International Union for Conservation of Nature
KMGBF: Kunming-Montreal Global Biodiversity Framework
SBTN: Science-based Targets Network
SME: small- and medium-sized companies
STAR: Species Threat Abatement and Restoration
TNFD: Taskforce for Nature-related Financial Disclosure
